# Hypoxia Induced NF-κB

**DOI:** 10.3390/cells5010010

**Published:** 2016-03-08

**Authors:** Laura D’Ignazio, Sonia Rocha

**Affiliations:** Centre for Gene Regulation and Expression, School of Life Sciences, University of Dundee, Dow street, Dundee DD1 5EH, UK; l.dignazio@dundee.ac.uk

**Keywords:** Hypoxia, NF-κB, IKK, HIF-1, PHDs, FIH, TAK, ubiquitin

## Abstract

As Nuclear Factor-κB (NF-κB) is a major transcription factor responding to cellular stress, it is perhaps not surprising that is activated by hypoxia, or decreased oxygen availability. However, how NF-κB becomes activated in hypoxia is still not completely understood. Several mechanisms have been proposed and this review will focus on the main findings highlighting the molecules that have been identified in the process of hypoxia induced NF-κB. In addition, we will discuss the role of NF-κB in the control of the cellular response to hypoxia.

## 1. Introduction

Hypoxia, or a decrease in oxygen availability, is both a physiological and pathological stimulus for cells [1]. Different tissues are exposed to different oxygen tensions, and when the demand for oxygen exceeds its supply, hypoxia ensues. This can be the case following tissue wounding, ischeamic stroke or intense metabolic activity, such as the one encountered following pathogenic related infections associated with increased macrophage infiltration. Hypoxia induces a comprehensive transcriptional program that involves the activation of the transcription factor Hypoxia Inducible Factor family (HIF). HIF is responsible for the induction of numerous genes involved in many different processes such as angiogenesis, cell proliferation, cell death and autophagy [2,3]. Oxygen sensitivity is achieved by a class of enzymes that are dioxygenases, called prolyl-hydroxylases (PHDs) and factor inhibiting HIF (FIH). PHDs regulate HIF stability via the association of the ubiquitin ligase complex containing the tumour suppressor von Hippel Lindau (VHL) [4], while FIH controls HIF association with the key coactivators p300 and CREB Binding Protein (CBP), thus regulating HIF’s transcriptional activity [5]. However, the cellular response to hypoxia does not only rely on HIF, as many other transcription factors have been shown to respond to hypoxia [2,6]. One of these is the family of transcription factors Nuclear Factor-κB (NF-κB). In this review we will focus on the pathways described for the activation of NF-κB in hypoxia and describe the roles of NF-κB in the cellular response to low oxygen. Of note, an extensive crosstalk exists between HIF and NF-κB, which has been recently reviewed elsewhere [7,8,9].

## 2. Pathways for NF-κB Activation, Canonical, Non-Canonical and Atypical

NF-κB is a family of transcription factors that is composed by RelA, RelB, cRel, NF-κB1 (p105/p50) and NF-κB2 (p100/p52) [10]. These transcription factors are usually held captive in the cytoplasm by a family of inhibitors called Inhibitor of κB (IκBs) [10]. Upon stimulation by different stresses, a number of pathways have been described that lead to nuclear accumulation and DNA-binding of NF-κB [11]. These pathways, can be subdivided in canonical, non-canonical and atypical pathways. The most well understood pathway is the canonical or classical pathway of NF-κB activation, involving activation of Transforming Growth Factor-B activating kinase (TAK1) and the Inhibitor of κB kinase complex (IKK), composed of IKKα, IKKβ and IKKγ or NF-κB essential modulator (Nemo) [12,13]. The non-canonical mode of NF-κB activation relies on NF-κB inducing kinase (NIK) and IKKγ homodimer activation [12,13]. On the other hand, atypical pathways leading to NF-κB activation usually do not require the IKK complex and act directly on the IκBs [13].

NF-κB activation pathways are heavily regulated by changes in ubiquitination of several components [14]. In fact, the study of NF-κB activation pathways has propelled the discovery of the function of different ubiquitin chains as well as novel deubiquinase enzymes, such Lysine 63 (K63) ubiquitin and Cyld [15], linear ubiquitin and otulin [16]. Furthermore, a study, investigating how Tumour Necrosis Factor (TNF) receptor engagement triggers the NF-κB activation pathways, identified a multitude of different ubiquitin chains present at the receptor, including K11 and K48 [17].

## 3. Activation of NF-κB by Hypoxia, Role of PHDs and FIH

**Figure 1:**
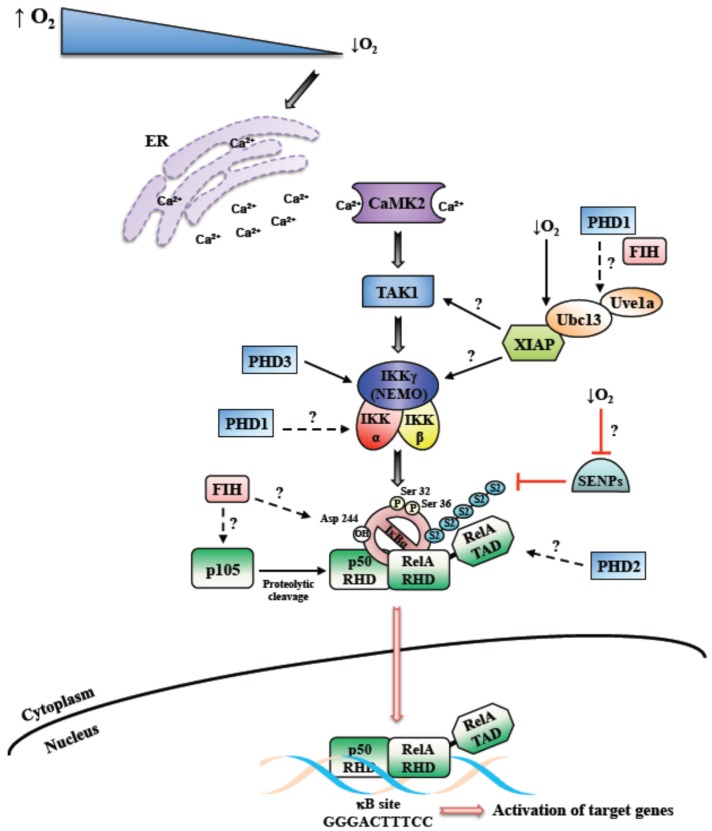
Mechanism of hypoxia induced Nuclear Factor-κB (NF-κB). Schematic diagram depicting the overview of the mechanism behind hypoxia induced NF-κB. Hypoxia induced NF-**κ**B has been shown to require Calcium, Transforming Growth Factor-B activating kinase (TAK1) and Inhibitor of κB kinase complex (IKK). In addition, contributions from dioxygenases such as prolyl-hydroxylases (PHDs) and factor inhibiting HIF (FIH) have been reported. Unanswered questions into the mechanisms of hypoxia induced NF-κB are highlighted as questions marks. SENPs-Sentrin/SUMO specific proteases; S2-Sumo-2.

For the activation of most pathways leading to NF-κB, a ligand is usually required to bind to a receptor, such as a cytokine or recognition of foreign DNA or RNA [18]. However, a stimulus such as DNA damage activates NF-κB by sensor kinases in the nucleus culminating in the activation of cytoplasmic IKK [19]. As such, how hypoxia induces NF-κB is far from clear. As mentioned above, for the activation of HIF, the cell relies on oxygen sensors such as PHDs and FIH [20]. Intuitively, these enzymes could provide the mechanism by which other signaling pathways in the cell are activated by reduced oxygen availability. A seminal study by the group of Cormac Taylor, suggested that indeed PHDs could link oxygen sensing to NF-κB activation, by directly impacting on IKKs themselves [6]. Here, PHD1 was identified as the isoform with the higher level of control over IKK activity (Figure 1). Since then, several other studies identified a role for PHDs in the control of NF-κB, however whether this requires the hydroxylase activity of the enzyme has received conflicting reports.

While it is clear that in certain cell types and/or tissues, PHD activity inhibition can lead to NF-κB activation [6,21], other studies suggested that PHDs might just act as a bridge between IKKγ and the ubiquitination machinery [22]. Furthermore, while it is not firmly established if PHDs hydroxylate any of the IKKs in the cell, hydroxylation sites have been found in ubiquitin ligases Uve1a and Ubc13, two enzymes required for NF-κB activation following Interleukin 1β (IL-1β) stimulation [23]. However, additional studies are required to identify the functional role of these modifications, in terms of their effects in the activation of NF-κB in hypoxia. Interestingly, our group had identified these enzymes as being required for hypoxia induced NF-κB [24,25].

More recently, PHD2 has been shown to act as a RelA co-activator, however, it is not clear if its enzymatic activity is required for such actions [26]. As such, additional studies designed to uncover hydroxylation sites and their functional roles are necessary to consolidate the theory that activation of the NF-κB pathway in hypoxia is mediated by PHDs.

Perhaps the best connection between hydroxylase activity and NF-κB is focused on FIH. Proteomic studies leading to the identification of new FIH targets, identified a number of components of the NF-κB pathways as being hydroxylated, including IκBs and NF-κB1 and NF-κB2 [27]. More recently, a study revealed potential targets in the upstream pathway, in particular in de-ubiquitinase enzyme OTUB1 [23,28]. Despite all of these targets, the functional significance of FIH-dependent hydroxylation on these proteins’ function remains unclear, as mutations of the acceptor asparagines have revealed very little information, as to whether they are important or not [27,29]. The exception to this, is hydroxylation of OTUB1 by FIH, where mutation of the acceptor asparagine changes the interactome of OTUB1 and gives rises to metabolic changes in the cell [28]. However, the importance of OTUB1 for the activation of NF-κB in hypoxia has not been demonstrated. As such, additional work is required to fully delineate the role of these dioxygenases in the control or activation of the pathways leading to NF-κB activity.

## 4. Activation of NF-κB by Hypoxia, Role of TAK and IKK

As mentioned above, to ensure a rapid NF-κB response, NF-κB subunits are not synthetized *de novo*, but kept inactive in the cytoplasm and, when necessary, activated by different signaling pathways. With the exception of the IKK-independent atypical pathways [30], the IKK kinase complex is a key element in the NF-κB signaling cascade. In this complex, IKKα and IKKβ are the two catalytic kinase subunits. Both of them are physiologically essential as knockout mice are, respectively, neonatal or embryonic lethal [31].

When, in 1994, it was reported for the first time that hypoxia can activate NF-κB, an IKK-independent mechanism of induction was hypothesized (see below) [32]. Although a number of studies followed that pivotal finding, additional mechanistic insights into NF-κB activation following hypoxia were lacking for another decade. We, and others, uncovered a potential mechanism regulating hypoxia-induced first wave of NF-κB activation, where IKK has a crucial role [24,33] (Figure 1). Our work showed that hypoxia leads to the NF-κB activation very rapidly, varying from 5 to 30 min. The important role of IKK was unveiled by multiple investigating approaches. Both in cancer cells and primary cells, the inhibition or deletion of IKK caused, respectively, partial or complete reduction of hypoxia-induced phosphorylation of IκBα on Serine 32 and 36. Furthermore, also the NF-κB DNA binding was affected [24,25]. These results were also conserved in the model organism *Drosophila melanogaster*, where hypoxia activates NF-κB in a IKK-dependent manner [34]. In this study, Ird5 (the fly IKK homologue) was found to be crucial to allow the transcription of NF-κB target genes activated by hypoxia.

In addition, we found that Transforming Growth Factor Activating Kinase 1 (TAK1), a member of the mitogen-activated protein kinase (MAPK) family, was required for IKK activation in this context (Figure 1). This role of TAK1 was already known after treatment with different stimuli, such as TNF-α, IL-1β and lipopolysaccharide (LPS) [35]. A number of evidences confirmed the critical importance of TAK1 also for the hypoxia-induced NF-κB activation via IKK, ultimately reflecting in the induction of specific NF-κB-dependent target gene expression. Among the known upstream factors involved in TAK1 activation, we found that Ca^2+^ release and consequent calcium/calmodulin-dependent kinase 2 (CaMK2) activity are required to obtain a functional activation of the NF-κB pathway in response to hypoxia (Figure 1). In addition, we showed that the E2 ubiquitin-conjugating enzyme Ubc13 is necessary, indicating that K63 ubiquitin chains are important for hypoxia induced NF-κB. In fact, Ubc13 expression was found to be increased in hypoxia, and specific Ubc13 depletion prevented IKK and IκBα phosphorylation [24]. We also identified XIAP as one of the possible E3-ligase interacting with Ubc13, essential for the induction of hypoxia-activated NF-κB pathway [25]. Both Ubc13 and XIAP are known to be required for K63-linked ubiquitin chains [36] (Figure 1).

Although in *Drosophila melanogaster*, the roles of Ca^2+^ and TAK1 have not been investigated, Cyld was identified as a negative regulator of hypoxia-induced NF-κB activation. In mammalian cells, Cyld is a NF-κB target, as well as a negative regulator of IKK activity [37], however its role in the hypoxia response has not been completely elucidated yet. However, our findings are in accordance with a previous study conducted by An and colleagues [38]. Specifically, they described Cyld as mediator of NF-κB activation after prolonged hypoxia, in cells expressing the E6 viral protein. These studies further highlight the role of K63-ubiquitin chains in hypoxia induced NF-κB.

Thus, TAK1-IKK axis seems to be the major mechanism underlying the activation of the NF-κB pathway following hypoxia stimuli, also taking into consideration its evolutionary conservation. However, the existence of other cell/tissue specific regulatory mechanisms activated in particular conditions, such as prolonged hypoxia, is still possible and further studies are needed.

## 5. Activation of NF-κB by Hypoxia, Modulation of IκB

In the cytoplasmic signaling cascade activated by hypoxia to induce NF-κB, IκBα has primary importance. This is one of the five cellular proteins recognized as NF-κB inhibitors. IκB family members, through their ankyrin repeats, are able to bind and mask the nuclear localization signal of NF-κB subunits, keeping them inactive into the cytoplasm [39].

As previously mentioned (see above), in the first study showing the NF-κB activation by hypoxia [32], a prominent role of a tyrosine phosphorylation of IκBα, involved in the dissociation of this inhibitory protein, as well as in the NF-κB DNA binding, was reported. In contrast to canonical NF-κB activation, characterized by IκBα degradation, hypoxia-induced NF-κB activation is not characterized by IκBα degradation. As such, an IKK-independent mechanism of activation was suggested. However, we, and others, demonstrated the crucial role played by IKK in the regulation of hypoxia-induced NF-κB [24,33,34]. Phosphorylation of IκBα on Ser32/36 is an important characteristic of the activation of the pathway via TAK1-IKK, on the other hand, no role for Tyrosine 42 (Y42) of IκBα could be identified, when mutants where analysed [24].

Nevertheless, IκBα is not degraded after hypoxia induction, as hypoxia prevents its ubiquitination. Particularly, the lack of IκBα degradation depends on its conjugation with Sumo-2/3 of Lysine 21 [24]. While Sumo-1 conjugation of IκBα has been linked to NF-κB inhibition [40], Sumo-2/3 conjugation, induced by hypoxia, seems to contribute to NF-κB activation (Figure 1). Specifically, our data suggested that hypoxia might prevent the activation of Sumo proteases, thus preventing desumoylation of IκBα [24,25]. Thus far, different models regulating IκBα sumoylation have been discovered in various contexts [41,42]. Interestingly, phosphorylated and sumoylated IκBα has been shown to be nuclear and regulate transcription mediated by polycomb [43]. Doubtless, further studies focused on sumoylated IκBα and sumo protease activity during the hypoxia response will be informative to complement the current knowledge.

## 6. Role of NF-κB in the Cellular Response to Hypoxia

Despite the mechanisms leading to NF-κB activation in hypoxia being still under investigation, it is now clear that indeed NF-κB responds to this stimulus. As such, the functional consequences of this activation also need to be investigated. For the majority of studies investigating cancer cells, NF-κB activation following hypoxia results in decreased apoptosis and increased angiogenesis [24,44,45,46]. However, the situation is different in tissues such as the brain and the heart, where NF-κB activation seems to have a more complicated role both inducing and repressing apoptosis depending on context [47].

The functional consequences of hypoxia induced NF-κB has been mainly studied in disease models. One major response controlled by NF-κB under this stimulus is cell death. Under hypoxia, NF-κB has been shown to modulate the expression of numerous proteins involved in the control of apoptosis such as Bcl-2 family members as well as inhibitors of apoptosis [24,48,49]. As such hypoxia induced NF-κB is important for the survival programme in cancer.

In addition, hypoxia induced NF-κB has been shown to upregulate the expression of IL-8 [24], an important chemokine for the induction of angiogenesis [50], thus contributing to the overall neo-vasculature generation occurring in hypoxia.

Hypoxia induced NF-κB has also been shown to induce the expression of protein involved in motility and adhesion such as matrix metalloproteases (MMPs) and SDF-1 [51,52,53]. These have been seen in cancer cell lines but also in immune and neuronal cells in conditions such arthritis and stroke [51,52,53].

In conclusion, hypoxia induced NF-κB is important for many of the cellular responses elicited by this stimulus, in particular for prevention of apoptosis, induction of angiogenesis and promoting motility of cells.

## 7. Conclusions

At least in some cell types, hypoxia is a relatively mild (yet robust) activator of the NF-κB response in comparison to, for example, cytokine stimulation, and this may have implications for which set of target genes are activated by NF-κB in hypoxia. Hypoxia induced NF-κB is IKK dependent and relies on the canonical signaling cascade for this activation (Figure 1). However, the involvement of dioxygenase such as PHDs and FIH has been reported and these might have important roles in the control of the NF-κB pathway under different cellular contexts and following prolonged hypoxia exposures (Figure 1). It is therefore likely that hypoxia induced NF-κB is multifaceted and may well involve several or all of the mechanisms mentioned above. Additional studies are required to answer the remaining questions concerning the mechanisms of NF-κB activation in hypoxia (Figure 1).
